# Psychometric properties of the Inventory of Life Quality in children and adolescents in Norwegian Sign Language

**DOI:** 10.1186/s40359-021-00590-x

**Published:** 2021-05-27

**Authors:** Chris Margaret Aanondsen, Thomas Jozefiak, Kerstin Heiling, Stian Lydersen, Tormod Rimehaug

**Affiliations:** 1 Unit for Deaf and Hard-of-Hearing Children and Adolescents in Central Norway, Department of Child and Adolescent Psychiatry, St. Olavs Hospital Trondheim University Hospital, Trondheim, Norway; 2 Department of Mental Health, Faculty of Medicine and Health Sciences, NTNU—Norwegian University of Science and Technology, Trondheim, Norway; 3 Department of Child and Adolescent Psychiatry, IKVL, Psychiatry Skåne, Lund University, Lund, Sweden; 4 Department of Child and Adolescent Psychiatry, Nord-Trøndelag Hospital Trust, Levanger, Norway

**Keywords:** Quality of life, DHH children, Psychometric properties, Sign language

## Abstract

**Background:**

Several studies have assessed the Quality of Life (QoL) in Deaf and hard-of-hearing (DHH) children and adolescents. The findings from these studies, however, vary from DHH children reporting lower QoL than their typically hearing (TH) peers to similar QoL and even higher QoL. These differences have been attributed to contextual and individual factors such as degree of access to communication, the participants’ age as well as measurement error. Using written instead of sign language measures has been shown to underestimate mental health symptoms in DHH children and adolescents. It is expected that translating generic QoL measures into sign language will help gain more accurate reports from DHH children and adolescents, thus eliminating one of the sources for the observed differences in research conclusions. Hence, the aim of the current study is to translate the Inventory of Life Quality in Children and Adolescents into Norwegian Sign Language (ILC-NSL) and to evaluate the psychometric properties of the self-report of the ILC-NSL and the written Norwegian version (ILC-NOR) for DHH children and adolescents. The parent report was included for comparison. Associations between child self-report and parent-report are also provided.

**Methods:**

Fifty-six DHH children completed the ILC-NSL and ILC-NOR in randomized order while their parents completed the parent-report of the ILC-NOR and a questionnaire on hearing- and language-related information. Internal consistency was examined using Dillon-Goldstein’s rho and Cronbach’s alpha, ILC-NSL and ILC-NOR were compared using intraclass correlation coefficients. Construct validity was examined by partial least squares structural equation modeling (PLS-SEM).

**Results:**

Regarding reliability, the internal consistency was established as acceptable to good, whereas the comparison of the ILC-NSL with the ILC-NOR demonstrated closer correspondence for the adolescent version of the ILC than for the child version. The construct validity, as evaluated by PLS-SEM, resulted in an acceptable fit for the proposed one-factor model for both language versions for adolescents as well as the complete sample.

**Conclusion:**

The reliability and validity of the ILC-NSL seem promising, especially for the adolescent version, even though the validation was based on a small sample of DHH children and adolescents.

**Supplementary Information:**

The online version contains supplementary material available at 10.1186/s40359-021-00590-x.

## Background

### Quality of life in  Deaf and hard-og-hearing children  and adolescents

The number of studies on Quality of Life (QoL) in Deaf and hard-of-hearing (DHH) children and adolescents has increased over the past decades, mainly focusing on children with cochlear implants. However, as Hintermair [1], points out, several aspects make it difficult to compare these studies. Among these are differences in the definition of QoL, ranging from Health-Related QoL (HRQoL) to social well-being, different types of assessments (generic QoL measures, ad-hoc tools designed for specific studies, and parents’ qualitative reports after their children’s cochlear implantation), and different informants (parents and children) as well as differences in access to communication and peers. Researchers such as Warner-Czyz et al. [2] have demonstrated the importance of including both parents’ and children’s perceptions. They found that 4–7-year-old DHH children in their study reported better QoL than their parents. Chmiel et al. [3] support this necessity based on parents reporting better QoL for their 3–20-year-old DHH children and adolescents after cochlear implantation when compared with their children’s self-report. Fellinger et al. [4] also report low agreement between parents and their 6–16-year-old DHH children and adolescents on the Inventory of Life Quality in Children and Adolescents (ILC). Parents report the same level of QoL for their DHH children as parents of a typically hearing (TH) normative sample. The DHH children themselves report being less satisfied with play/hobbies when alone, as well as physical health, compared with TH normative data. The same DHH children report better QoL related to school and family. Other researchers such as Pardo-Gijarro et al. [5], on the other hand, find moderate agreement between Spanish DHH children and adolescents and their parents when using a written and a Spanish sign language version of the KIDDSCREEN27, with correlations between 0.377 and 0.753. Discrepancies between child- and parent-report have also been reported for TH children and adolescents [6, 7]. Therefore, the multi-informant approach has been emphasized for accessing QoL. Other factors that are likely to have contributed to differences in DHH children and adolescents’ QoL are variations in participants’ age, their preferred mode of communication and degree of hearing loss. It has previously been found for both TH and DHH children that older adolescents report lower QoL [5, 8–10]. The development of reliable and valid QoL instruments in sign language will help gain more accurate reports from DHH children who use sign language as their preferred language**,** thus eliminating one of the sources for the observed differences in research conclusions. In the present study, the term “children” is used for those aged 11 and younger, whereas “adolescents” refers to those aged 12 and older.

In their systematic review Roland, Fischer, Tran, et al. [11] report that 11 of 16 studies based on DHH children and adolescents and validated QoL measures find significantly lower QoL when compared with normative scores or TH controls, whereas five studies do not identify such differences in QoL. Their meta-analysis reveals that DHH children and adolescents report decreased QoL in the social and school domains based on the Pediatric Quality of Life Inventory (PedsQL). Unfortunately, there are some issues with this systematic review [11]. One problem is the lack of information about the informants for the specific studies.

Another issue with Roland, Fischer, Tran, et al.’s [11] systematic review is that Hintermair’s [1] and Fellinger, Holzinger, Sattel, et al.’s [4] results are cited wrongly, that is, a maximum of 9 out of 16 studies (not 11 out of 16 as the authors state) find significantly lower QoL when compared with normative scores or TH controls. Hintermair [1] finds that mainstreamed DHH children and adolescents report better QoL based on the total QoL score, as well as in the domains of school, physical health, mental health, and global QoL, on the ILC than a normative TH sample. The effect sizes for the reported differences were small to moderate. Fellinger, Holzinger, Gerich, et al. [12] and Hintermair [1] report QoL being unrelated to the type and degree of hearing loss in DHH adults, children and adolescents respectively, whereas others such as Tsimpida, Kaitelidou, and Galanis [13] find that DHH adults with a higher degree of hearing loss report lower QoL. Kushalnagar, Topolski, Schick et al. [14] demonstrate that adolescents (11–18 years old) report higher QoL when they perceive that they understand most of their parents’ expressive communication. This was not dependent on their preferred communication modality or degree of hearing loss. Adolescents with a preference for a combination of sign language and speech, however, reported experiencing less stigma than those with a strong preference for speech only [14].

### Assessing QoL in DHH children and adolescents

Language and communication are essential for assessing QoL. Sign languages are natural languages that share many linguistic characteristics with spoken languages but also have specific features due to their manual-visual nature [15]. Studies have also shown that cultural context influences the understanding of seemingly identical wordings, especially when translating from written text to sign language [16, 17]. The acknowledgment of sign languages as natural languages has helped lead to a shift from viewing DHH people in a medical and disability perspective to a socio-cultural one, appreciating deaf culture with its language, history, traditions, art and values [18, 19]. For several DHH children and adolescents written language is considered as their second language. Studies have reported reading difficulties for many DHH children and adolescents [20–22], which in turn are likely to affect their ability to complete written forms, compromising the validity of assessments based on written forms. When assessing symptoms of mental health problems in DHH children and adolescents, it has been confirmed that the use of written self-report measures can lead to underestimating symptoms [23, 24]. Most measures are designed for assessing TH people. A common solution in clinical practice is the use of sign language interpreters, who will provide on-the-spot translations, which will be influenced by their training and experience and therefore vary across settings and children [25]. Pardo-Guijarro, Martínez-Andrés, Notario- Pacheco et al. [5] emphasize the need to translate valid and reliable generic QoL measures into sign language to assess QoL in DHH children and adolescents and compare them to their TH peers’ QoL. Assessment tools for QoL exist in some sign languages so far—American [26], Austrian [27], and Spanish Sign Language [5]. To the best of our knowledge, there is a lack of such instruments and a lack of studies on QoL in Norwegian DHH children and adolescents.

### The Inventory of Life Quality (ILC)

The ILC is a brief measure to assess QoL in children and adolescents. The measure is based on the concept of the individual’s perception of their position in life, including their health, functioning, and participation in routines and activities as compared to their peers [6, 7]. It consists of seven items. One item for Global QoL and six items addressing the child’s physical and mental health, school and family functioning, social contact with peers as well as play/hobbies when alone. The ILC is a multi-informant assessment and can be completed by children, adolescents, and young adults aged 6–21 and their parents. For children aged 6–11, the self-report is administered as an interview. Achenbach, McConaughy and Howell [28] among others, emphasize the importance of multi-informant assessments for capturing the unique perspectives held by each informant.

The original German validation found acceptable internal consistency (α = 0.63 self-report and α = 0.76 parent report) and test–retest reliability (r = 0.72 self-report and r = 0.80 parent report) for the QoL score (LQ_0–28_) for community samples. Convergent validity with the Kinder Lebensqualität Fragebogen (KINDL) was shown to be moderate. Construct validity based on Principal Component Analysis was found to be acceptable for the one-component model in a community sample (self- and parent-report; N = 9292 and N = 1109) and a two-component model in a clinical sample (self- and parent-report; N = 605 and N = 568) [7]. For the two-component model, one component consisted of one item only (play/hobbies when alone) and the other component of the other six items. Based on the low number of items as well as the nature of the clinical sample and the relatively lower number of participants, the authors concluded that the one-component model fit the theoretical model best [7]. The importance of examining psychometric properties for measures of QoL in both community and clinical samples has been demonstrated by Jozefiak, Mattejat and Remschmidt [6] amongst others when examining the relationship between depression and QoL.

The validation of the Norwegian self and parent report [6] found satisfactory internal consistency for adolescents aged 11 and older (self-report: Cronbach’s α = 0.80–0.82, parent report: α = 0.78). For children aged ten and younger, internal consistency was somewhat lower (α = 0.64). The two-week test–retest reliability for the self-report was found to be high (r = 0.86). The one-factor model of the ILC based on confirmatory factor analysis demonstrated good fit in three community samples and acceptable fit in the fourth (clinical) sample. Moderate correlations between the KINDL and ILC self-report were found, supporting convergent validity [6]. A systematic Norwegian review based on five studies of the psychometric properties of the ILC confirmed these findings [29].

To the best of our knowledge, the ILC has only been used to study QoL in DHH in Germany, Austria, and Norway. Construct validity for DHH children and adolescents has only been studied in Germany [1]. In this sample, the DHH children and adolescents were all mainstreamed, indicated a preference for spoken language, and were assessed with the original written version. Hintermair [1] finds satisfactory internal consistency (α = 0.71) for the ILC in this German DHH sample with 212 participants; interitem correlations showed the same pattern as for TH children and adolescents with the items “Mental Health” and “Global QoL”, demonstrating the highest correlations with the QoL score (LQ_0–28_). A principal component analysis with subsequent varimax rotation resulted in the best fit for the two-component  solution, “Family” and “Alone (play/hobbies),” constituting one component, while the other five items constituted the other component. Hintermair [1] concludes that these results support the use of the ILC for DHH mainstreamed children and adolescents with a preference for spoken language.

Except for the pilot study by Aanondsen et al. [8], there are hardly any studies on Norwegian DHH children and adolescents’ QoL, and no studies validating assessment tools in NSL for assessing QoL in DHH children and adolescents. Norway is unique in offering the parents of DHH children and adolescents 40 weeks (i.e., 2–4 weeks/year) of NSL classes over the course of 16 years, with all expenses covered. Therefore, one might expect a higher level of sign language skills among Norwegian DHH children and adolescents and their parents. This, in turn, may have a positive influence on their QoL. The inconsistencies in previous studies regarding DHH children and adolescents’ QoL necessitate valid tools, both written and in sign language, to bridge the gap. The present study contributes to this by both translating the ILC to NSL as well as providing psychometric properties for the Norwegian version of the ILC self-report (ILC-NOR) and the NSL version (ILC NSL). The ILC NSL is the first instrument translated to NSL for assessing QoL in Norwegian DHH children and adolescents.

## Methods

### Aims

The main aims of the present study were to translate and validate the ILC self-report in NSL (ILC-NSL) and compare it with the ILC-NOR in Norwegian DHH children and adolescents. Both self-reports of the ILC were compared with the parent report. Finally, the usability of the ILC-NSL for signing DHH children and adolescents was assessed from the children and adolescents’ perspective.

We addressed the following research questions.

1. What is the internal consistency of the ILC-NSL and ILC-NOR for DHH children and adolescents?
2. What are the correlations between the total scores and items between the self-report ILC-NSL and ILC-NOR?
3. What is the construct validity of the ILC-NSL and ILC-NOR for DHH children and adolescents?
4. What are the correlations between the QoL score (LQ_0–28_) and items between the self-reports (ILC-NSL and ILC-NOR) and parent report?
5. What do DHH children and adolescents think about the usability of the ILC-NSL and ILC-NOR?

### Participants

Caluraud, Marcolla-Bouchetemblé, de Barros et al. [30] report that hearing loss (HL) of > 40 dB affects 1.4 per 1000 infants (mild HL in 13%, moderate HL in 50%, severe HL in 17%, and profound HL in 20%). In central and northern Norway, this amounts to 266 children and adolescents with a HL of > 40 dB, that is, 45 with severe and 53 with profound HL based on a population of 189,737 children and adolescents aged 6–18.

**Fig. 1:**
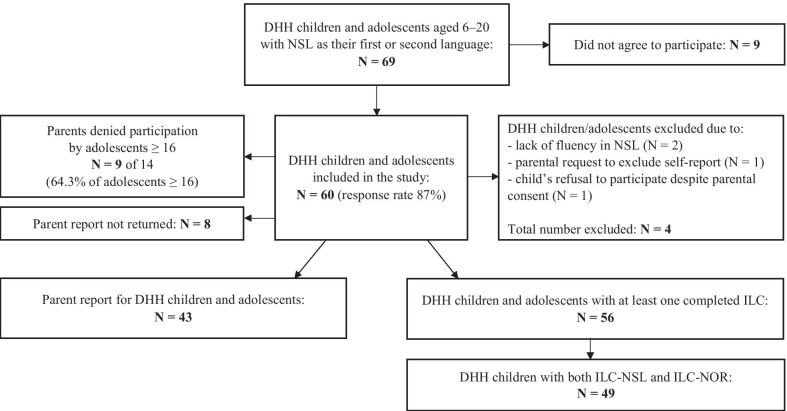
Flow chart for the inclusion of participants

DHH children and adolescents aged 6–17 were recruited from the part- and full-time students at A.C. Møller school, a Deaf school for central and northern Norway during the school year of 2016/17. DHH adolescents aged 15–20 attending Tiller upper secondary school in central Norway with NSL as their first or second language were also invited. The overall response rate for the combined subsamples was 87% (60/69) (see Fig. 1).

Two children were excluded because of a lack of fluency in Norwegian sign language. Apart from fluency in both written and signed Norwegian (NSL), we applied no exclusion criteria. In total, 56 DHH children and adolescents (67.9% girls) participated in the current study, with a mean age of 12.4 years (SD = 3.65; range = 6–20) and a mean nonverbal IQ of 106.91 (SD = 17.74; range = 49–143). The participant with the lowest non-verbal IQ was included in further analysis despite being an extreme outlier in the IQ distribution (range excluding outlier = 74–143). Thirty-seven of the 42 (88.1%) mothers had completed 12 years or more of education, whereas 28 of the 41 (68.3%) fathers had completed 12 years or more of education. Data were collected between November 1, 2016 and May 9, 2017. The majority of the DHH children and adolescents (69%) mainly attended mainstream schools while spending two to six weeks at the deaf school per school year.

Hearing- and language-related information for the participants in the current study can be found in Tables 1 and 2.

**Table 1:** Hearing-related characteristics (parent report)

Variable	N = 42	%
DHH family member(s)		
Yes/no	22/20	52.4/47.6
Time in deaf school ^a^		
1–2 days a week ^b^	8	19.0
5 days a week	4	9.5
2–6 weeks a year	29	69.0
> 7 weeks a year	8	19.0
Etiology of hearing loss		
Acquired	4	9.5
Hereditary/at birth	36	85.7
Unknown	1	2.4
Missing	1	2.4
Severity of hearing loss		
Moderate: 40–70 dB	10	23.8
Severe: 71–100 dB	14	33.3
Profound: 101+	12	28.6
Unknown	5	11.9
Missing	1	2.4
Use of hearing aid (yes/no) ^c^		
CI	20/21	47.6/50.0
Hearing aid	33/ 8	78.6/19.0
Missing	1	2.4
Age at diagnosis		
0–2 years	27	64.3
3–5 years	15	35.7
Preferred language		
Oral	21	50.0
Sign	6	14.3
Bilingual	15	35.7
Other impairment		
Vision	14	32.6
Motor	1	2.3
Learning	4	9.3
Other	8	18.7
Missing	3	7.0

^a^All children attend both mainstream and deaf school

^b^Children attending the deaf school for 1–2 days a week combine this with two or more week-long stays during the school year; that is, total number of answers is greater than the number of participants

^c^Based on reports of ever having used a hearing aid

**Table 2:** Language-related information based on parent report

Language skills	N	M (SD)
Sign language skills (1–12; CA)	38	9.05 (2.09)
Ages 6–11 (ILC Child)^1^	23	8.87 (1.82)
Ages 12–20 (ILC Adol.)	15	9.33 (2.50)
Spoken language skills (1–12; CA)	40	11.20 (1.70)
Ages 6–11 (ILC Child)^1,2^	25	11.28 (1.70)
Ages 12–20 (ILC Adol.)	15	11.07 (1.75)

Sign Language Skills based on the sum scores of the sign language production scale (SPS) and the sign language understanding scale (SUS); range 0–12. Higher scores indicate better communication skills

Spoken Languages Skills based on the sum scores of Categories of Auditory Performance (CAP) and Speech Intelligibility Rating (SIR); range 0–12. Higher scores indicate better communication skills

*CA* children and adolescents—complete sample

^1^Significant difference between scores for Sign Language Skills (M = 8.87, SD = 1.82) and Spoken Language Skills (M = 11.28, SD = 1.70) for ages 6–11; t(22) =  − 5.53, p < .001

^2^One extreme outlier with a sum score of 4 (> 3 SD) was found for ages 6 to 11 for spoken language skills

### Measures

#### Sociodemographic and hearing-related information

A questionnaire completed by the parents was used to assess the participants’ age, sex, type and severity of HL, type of education, and parents’ attendance of sign language classes. The same questionnaire was also used in a previous study by the same authors [31].

#### Language-related information

##### Spoken language skills

Categories of Auditory Performance (CAP; Archbold, Lutman and Marshall [32]) and Speech Intelligibility Rating (SIR, Allen, Nikolopoulos, Dyar et al. [33]) were used to assess participants’ speech intelligibility and listening skills. The CAP is a single-item scale with a range of 0–7. Level 0 is “no awareness of environmental sounds”, and Level 7 “uses a telephone with a known speaker.” The SIR is also a single-item scale with a range of 1–5. Level 1 is “connected speech is unintelligible”, and 5 “connected speech is intelligible to all listeners.” The interrater reliability of the Danish version is based on the reports of two teachers and was reported as good (CAP: kappa = 0.785; SIR: kappa = 0.848; Dammeyer [34]). The Norwegian versions of the CAP and SIR were recently used in a study by Aanondsen, Jozefiak, Heiling et al. [31] for a similar group of participants. The scores of CAP and SIR were combined to form the Spoken Language Skills Score.

##### Sign language skills

The Norwegian versions of the Sign Language Production Scale (SPS) and the Sign Language Understanding Scale (SUS) were used to assess Sign Language Skills [34]. The SPS and SUS were designed as as a short screening of sign language skills for research purposes and have previously been used in Norway [31]. SUS and SPS are based on the structure and range of CAP and SIR. The SPS is a single-item scale with a range of 1–5. Level 1 is “the child does not produce real signs” and Level 5 “the child uses fluent and almost conventional correct sign language.” The SUS is a single-item scale with a range of 0–7. Level 0 is “does not react to or does not comprehend signs” and Level 7 “is able to participate in long and complex conversations in sign language.” The interrater reliability of the Danish version based on the reports of two teachers was reported as good (kappa = 0.944 for SUS and kappa = 0.921 for SPS; Dammeyer [34]). The Danish version [35] of Assessing British Sign Language Development: Receptive Skills Test [36] was used to assess the validity of the SUS. The SUS and the sign language receptive skills test correlated significantly (Spearman rank correlation coefficient = 0.905, p < 0.000; [37]). The validity of the SPS could not be evaluated due to the lack of a comparable assessment. The scores of SPS and SUS were combined to form the “Sign Language Skills Score”.

#### Cognitive abilities

The Leiter International Performance Scale – Third Edition (Leiter-3) was used to assess nonverbal intelligence. It includes the following subtests: Figure Ground, Form Completion, Classification/Analogies, and Sequential Order. The sum of the scaled scores for these subtests constitutes the composite score of nonverbal IQ and is converted to the standard score [38].

#### Quality of life (QoL)

The Inventory of Life Quality in Children and Adolescents—ILC [6, 7] is a multi-informant assessment for QoL based on seven items. One item assesses overall QoL, and six items address the child’s physical and mental health, school and family functioning, social contact with peers, play/hobbies when alone. Items are rated on a 5-point Likert scale from 1 = “Very Good” to 5 = “Very Bad.” The QoL score (LQ_0–28_) is calculated by multiplying the mean of the seven items by seven and subtracting 35, thus obtaining absolute values with a range of 0 to 28; higher scores representing better QoL (LQ_0–28_) and lower QoL scores reflecting poorer overall QoL [6, 7].

In the current study, we administered the written parent report (ILC-NOR) and the self-report versions for children (6–11) and adolescents (12 and older) in both written and signed Norwegian (ILC-NOR and ILC-NSL), according to the manual [6]. Because of the differences reported [6] in internal consistency between the adolescent (Cronbach’s α = 0.81) and the child version (Cronbach’s α = 0.64), psychometric properties will be reported separately for the child and the adolescent versions, as well as for the complete sample (CA).

### The translation process

The translation of the ILC was conducted based on the guidelines for cross-cultural adaptation of written self-report measures by Beaton, Bombardier, Guillemin et al. [39] with adaptations suggested by Roberts, Wright, Moore et al. [25]. Suggestions were based on the differences in syntax, morphology and prosody of sign languages and their visual nature. The same translation process was applied and described in this study by Aanondsen, Jozefiak, Heiling et al. [31]. The ILC-NOR went through two independent forward and backward translations from written Norwegian to NSL. Two bilingual deaf native NSL users with university degrees in teaching conducted and recorded these. The semantic, conceptual, lexical, and cultural differences were discussed by a panel. Members of the panel were the translators, a clinical psychologist, a colleague with a graduate degree in medicine specializing in child and adolescent psychiatry, and a consultant with a master’s degree in language and communication and fluency in NSL. Based on these discussions, the panel developed a consensus-based forward translation that was filmed. Teachers from the local deaf school were used as a focus group. Best practice recommends including DHH children and adolescents in these focus groups. Due to constraints related to time and access to children of the right ages, teachers, who meet DHH children and adolescents with varying degrees of NSL and ages were recruited instead. The teachers (Deaf, hearing, and CODA, that is, a TH person raised by deaf parents) were asked to evaluate whether DHH children and adolescents with a mixture of language experiences and levels of fluency would be able to understand the translation. Based on the feedback of the focus group, the consensus version was adjusted and filmed again. Two hearing sign language interpreters, one with a background as a CODA and a master’s degree in language and communication conducted the backward translations of the final consensus version. These were reviewed by the panel and compared with the original written Norwegian version.

The author of the Norwegian version of the ILC, Thomas Jozefiak, approved the items and made suggestions for those not approved on behalf of the copyright holders (Hogrefe). These items went back through the translation cycle until final approval was achieved. After the final approval, the ILC-NSL was filmed professionally and prepared for interactive online administration using Select Survey.

### Procedures

The enrolled children and adolescents and their parents received oral/signed and written information about participating in the study during their first attendance at the school after the survey had been initiated. Written informed consent was obtained from the adolescents and parents prior to inclusion, according to the study’s survey procedures. The participating children and adolescents responded to the web-based ILC-NSL and ILC-NOR as well as a question about the usability of the two language versions and completed a nonverbal cognitive assessment. The nonverbal cognitive assessment was administered by a psychologist experienced in working with DHH children in mental health services and fluent in NSL. The administration of the ILC-NSL and ILC-NOR were conducted on two separate occasions with an interval of two to three days. The order of these two administrations was randomized. Parents also responded to a questionnaire on socioeconomic status, as well as questionnaires assessing their children’s mental health, communication skills in spoken and signed Norwegian, and hearing status. DHH children and adolescents had access to their teacher and a psychologist, both of whom were fluent in NSL, during data collection. When the children and adolescents asked for help with the ILC-NSL, they received support in NSL, whereas the children and adolescents replying to the ILC-NOR were assisted in spoken Norwegian or sign-supported speech.

### Statistical analyses

Missing values on five cases with ≤ 3 missing item values were substituted using expectation maximization (EM; [40]). Gender differences in item and scale mean scores were analyzed using independent samples t-tests. Mean differences were calculated. Bootstrapped confidence intervals were calculated using the bias corrected and accelerated method (BCa) and B = 1000 bootstrap samples. Differences between spoken and sign language skills were analyzed using paired sample t-tests for both age groups.

Dillon –Goldstein’s rho (DG rho) was used to assess internal consistency because of the limitations of Cronbach’s α, such as assumptions of uncorrelated errors, tau-equivalence and normality [41]. As most authors, however, report internal consistency based on Cronbach’s α, we also calculated Cronbach’s α, including bootstrapped confidence intervals for comparison. DG rho and Cronbach’s α were interpreted as acceptable internal consistency at 0.6–0.7, and as good internal consistency when > 0.7. Intraclass correlation coefficients (ICC) based on a two-way mixed effects model with absolute agreement were used to evaluate associations between the scale and item scores of the two self-reports (ILC-NSL and ILC-NOR). Intraclass correlation coefficients (ICC) were calculated for each of the seven items and the QoL score LQ_0–28_ to compare the two language versions of the self-report. We calculated Spearman’s rank correlations to assess multi-informant correlations between the QoL scores on the parent and self-reported versions (NSL and NOR).

Partial least squares structural equation modeling (PLS-SEM) is a robust method when dealing with small sample sizes because it is nonparametric and makes fewer distributional assumptions. PLS-SEM, however, is mostly used for exploratory purposes because it lacks goodness of fit measures. Because of the small sample size, we primarily used PLS-SEM to establish factor loadings and discriminant validity (average variance extracted (AVE)) as suggested by Hair, Hult, Ringle et al. [42]. Standardized factor loadings greater than 0.4 were considered acceptable [43]. Factors with AVE scores greater than 0.5 were regarded as satisfactory for convergent/discriminant validity. Fornell and Larcker [44], however, argue that AVE > 0.4 can be treated as acceptable if composite reliability is above 0.6.

As a supplementary analysis of the confirmed ILC factor structure, we used confirmatory factor analysis (CFA) with the weighted least squares means and variances adjusted (WLSMV) estimation method for categorical variables. The chi-square test, the normed chi-square (χ^2^/df), the root mean square error of approximation (RMSEA), comparative fit index (CFI) and Tucker-Lewis Index (TLI) were used to assess model fit. A non-significant chi-square test, CFI and TFI > 0.9, RMSEA < 0.1 were considered indicators of acceptable goodness of fit according to Mehmetoglu and Jakobsen [43], whereas CFI and TFI > 0.95 and RMSEA < 0.05 were considered as indicators of good model fit [45]. A normed chi-square of < 2.0 was considered as good for this study, and ratios of < 5.0 as acceptable [46]. Standardized factor loadings greater than 0.4 were considered acceptable [43]. Hair, Hult, Ringle et al. [42] point out that a small sample size can cause problems with underidentified models and nonconvergence in CFA. The estimator WLSMV has been shown to overestimate interfactor correlations when the sample size is relatively small [47]. Due to these problems, the CFA was used as a supplementary analysis only and can be found in Additional file 1: Appendix C. All analyses were conducted separately for the child and the adolescent versions, as well as for the complete age sample, that is, both the child and adolescent versions combined (CA).

The CFA was conducted in *M*Plus version 8. All otheranalyses were conducted in Stata/SE 14.2 for Windows. PLS-SEM, including AVE, was conducted in Stata by applying the module for PLS-SEM [48]. For all analyses, two-sided p-values < 0.05 were considered statistically significant.

### Ethics

Written informed consent was obtained from the parents and adolescents older than 16 prior to inclusion, as well as oral/signed informed consent from the children and adolescents under the age of 16. Study approval was given by the Regional Committees for Medical and Health Research Ethics (reference number: 2015/1739/REK midt).

## Results

Table 3 presents the means and standard deviations for the DHH participants on the self-report of the ILC (ILC-NSL and ILC-NOR). A table with mean differences for all items and bootstrapped confidence intervals can be found in Additional file 1: Appendix A. The full distribution of all items and QoL score for both self-reports is reported in Additional file 1: Appendix B.

**Table 3:** Mean and SD for ILC-NSL and ILC-NOR self-report item scores and QoL Score (LQ_0–28_)

ILC	School	Family	Other children	Alone (play/hobbies)	Physical Health	Mental Health	Global QoL	LQ_0–28_
ILC-NSL CA^1^ (N = 49)	1.86 (0.95)	1.71 (0.86)	2.05 (1.05)	2.33 (1.07)	1.92 (0.86)	2.02. (1.05)	1.97 (0.81)	21.15 (4.23)
ILC-NSL child (N = 22)	1.59 (0.78)	1.76 (0.87)	1.94 (1.10)	2.64 (1.05)	1.95 (1.09)	1.81 (1.01)	1.75 (.81)	21.56 (3.99)
ILC-NSL adol. (N = 27)	2.07 (1.04)	1.67 (0.88)	2.15 (1.03)	2.07 (1.04)	1.89 (0.70)	2.19 (1.08)	2.15 (0.77)	20.81 (4.47)
ILC-NOR CA^1^ (N = 56)	1.89 (0.93)	1.45 (0.74)	1.79 (0.75)	2.11 (1.06)	1.95 (1.02)	1.96 (0.93)	1.88 (0.99)	21.98 (4.51)
ILC-NOR child (N = 25)	1.48 (0.71)	1.32 (0.69)	1.61 (0.69)	2.28 (1.10)	1.76 (0.93)	1.47 (0.82)	1.52 (1.05)	23.56 (4.02)
ILC-NOR adol. (N = 31)	2.23 (0.96)	1.55 (0.77)	1.94 (0.77)	1.97 (1.02)	2.10 (1.08)	2.35 (0.84)	2.16 (0.86)	20.71 (4.54)

The Inventory of Life Quality in Children and Adolescents (ILC): Range of item scores 1–5, 1 = high QoL; QoL score (LQ_0–28)_: range 0–28, 28 = high QoL

^1^*CA*: children and adolescents—complete sample

Independent sample t-tests for the complete sample showed a significant gender difference for the QoL score LQ_0–28_ (girls: M = 20.916, SD = 0.780; boys: M = 24.239, SD = 0.651); t(54) =  − 2.720, p = 0.009 for the ILC-NOR and none for the ILC-NSL.

### Reliability

#### Internal consistency

As can be seen in Table 4, internal consistency based on DG rho and Cronbach’s α was found to be good for all scales and age versions, except for the ILC-NSL child version, which demonstrated acceptable internal consistency based on Cronbach’s α and good internal consistency based on DG rho.

**Table 4:** Internal consistency for the ILC

ILC LQ_0–28_	DG	Cronbach’s α	[95% CI]^2^	
ILC-NSL CA ^1^ (N = 49)	.827	.747	.569	.842
ILC-NSL child (N = 22)	.815	.698	.379	.874
ILC-NSL adol. (N = 27)	.861	.805	.618	.949
ILC-NOR CA^1^ (N = 56)	.874	.824	.704	.903
ILC-NOR child (N = 25)	.856	.785	.491	.880
ILC-NOR adol. (N = 31)	.885	.842	.680	.923

The Inventory of Life Quality in Children and Adolescents (ILC); QoL score (LQ_0–28_)

^1^*CA* children and adolescents—complete sample

^2^ CI: bootstrapped confidence intervals

#### Comparison of the ILC-NSL and ILC-NOR

To compare the ILC-NSL with the ILC-NOR self-report, intraclass correlation coefficients (ICC) were calculated for each of the seven items and the QoL score (Table 5).

**Table 5:** ICCs^1^ between ILC-NSL and ILC-NOR self-report

	M (SD) ILC-NSL	M (SD) ILC-NOR	ICC	[95% CI]		P
LQ_0–28_ CA (N = 49)	21.15 (4.23)	21.86 (4.52)	.508	.269	.688	< .001
School	1.86 (0.95)	1.92 (0.93)	.598	.382	.751	< .001
Family	1.71 (0.86)	1.51 (0.77)	.470	.225	.660	< .001
Other children	2.05 (1.05)	1.88 (0.75)	.288	.013	.524	.021
Alone	2.33 (1.07)	2.10 (1.00)	.409	.152	.616	< .001
Physical Health	1.92 (0.86)	1.92 (0.95)	.392	.123	.606	.003
Mental Health	2.02 (1.05)	1.93 (0.90)	.554	.326	.722	< .001
Global QoL	1.97 (0.81)	1.88 (0.99)	.217	-.069	.469	.068
LQ_0–28_ child (N = 22)	21.56 (3.99)	23.23 (4.14)	.012	-.381	.414	.478
School	1.59 (0.78)	1.55 (0.74)	-.019	-.457	.409	.532
Family	1.76 (0.87)	1.36 (0.73)	-.003	-.377	.393	.507
Other children	1.94 (1.10)	1.70 (0.70)	.104	-.324	.496	.319
Alone	2.64 (1.05)	2.23 (0.97)	.081	-.318	.469	.350
Physical Health	1.95 (1.09)	1.82 (0.96)	.363	-.067	.677	.048
Mental Health	1.82 (1.01)	1.53 (0.85)	.290	-.125	.624	.086
Global QoL	1.75 (.81)	1.59 (1.10)	-.143	-.551	.300	.734
LQ_0–28_ adol. (N = 27)	20.81 (4.47)	20.74 (4.57)	.836	.671	.922	< .001
School	2.07 (1.04)	2.23 (0.97)	.817	.642	.912	< .001
Family	1.67 (0.88)	1.63 (0.76)	.867	.729	.937	< .001
Other children	2.15 (1.03)	2.04 (0.76)	.424	.055	.689	.013
Alone	2.07 (1.04)	2.00 (1.04)	.651	.365	.825	< .001
Physical Health	1.89 (0.70)	2.00 (0.96)	.441	.079	.701	.010
Mental Health	2.19 (1.08)	2.26 (0.81)	.712	.461	.858	< .001
Global QoL	2.15 (0.77)	2.11 (0.85)	.511	.163	.744	.003

The Inventory of Life Quality in Children and Adolescents (ILC); QoL score (LQ_0–28_); CA: children and adolescents—complete sample

^1^*ICC* intraclass correlation coefficients based on a two-way mixed effects model with absolute agreement

The ICCs between the LQ_0–28_ of the ILC-NSL and ILC-NOR were highly significant at p < 0.001 for the complete sample, as well as for the adolescent version, but not for the child version.. The items on the adolescent versions were all significantly correlated, moderately to strongly (0.441–0.867), while none of the items on the child versions correlated significantly.

### Validity

#### Construct validity

The standardized factor loadings and AVE of the one-factor model are displayed in Table 6 for the ILC-NSL and ILC-NOR.

**Table 6:** Factor loadings and AVE of the ILC-NSL and ILC-NOR based on PLS-SEM CA: (children, adolescents, complete sample)

	*λ* (PLS)							
	School	Family	Other children	Alone	Physical Health	Mental Health	Global QoL	AVE
ILC-NSL CA (N = 49)	.784	.814	.698	.430	.269	.665	.722	.427
ILC-NSL child (N = 22)	.889	.856	.791	.306	− .041	.525	.790	.449
ILC NSL adol. (N = 27)	.719	.829	.564	.690	.622	.731	.625	.473
ILC-NOR CA (N = 56)	.802	.613	.707	.531	.594	.822	.836	.504
ILC-NOR child (N = 25)	.788	.348	.857	.553	.460	.809	.842	.480
ILC NOR adol. (N = 31)	.769	.762	.546	.702	.641	.797	.822	.526

The Inventory of Life Quality in Children and Adolescents (ILC); AVE: average variance extracted

All factor loadings were above the recommended 0.4 for both adolescent versions and the complete sample. The factor loading for “Family” on the ILC-NOR child as well as those for “Alone” and “Physical Health” on the ILC-NSL child were lower than recommended. AVE was above the acceptable 0.5 for the ILC-NOR CA and ILC-NOR child. Fornell and Larcker [44], however, argue that AVE > 0.4 can be treated as acceptable if composite reliability, in this case, DG’s rho, is above 0.6. This was the case for the complete sample as well as the child and adolescent versions of both the ILC-NSL and the ILC-NOR. Supplementary analyses based on CFA support these findings and can be found in Additional file 1: Appendix C.

### Multi-informant correlations

Multi-informant correlations between the LQ_0–28_ scores of DHH children and adolescents and their parents on the self-report ILC-NSL and ILC-NOR are presented in Tables 7 and 8. Correlations between the self- and parent-reported QoL score (LQ_0–28)_ were not significant for any of the versions. There was a moderate correlation for LQ_0–28_ of the adolescent ILC-NSL and the parent ILC. Analysis of the multi-informant correlations at the item level did not demonstrate significant correlations for any of the versions.

**Table 7:** Spearman rank correlations for the LQ_0–28_ of the ILC-NSL self- and parent report

	M (SD) ILC-NSL	M (SD) parent ILC	Spearman’s rho	[95% CI]^2^		P
LQ_0–28_ CA ^1^ (N = 35)	21.55 (3.89)	22.17 (4.00)	.057	− .363	.359	.746
LQ_0–28_ child (N = 22)	21.56 (3.99)	22.45 (3.56)	− .245	− .629	.213	.271
LQ_0–28_ adol. (N = 13)	21.54 (3.89)	21.69 (4.79)	.511	− .411	.911	.075

The Inventory of Life Quality in Children and Adolescents (ILC); QoL score (LQ_0–28_)

^1^*CA* children and adolescents—complete sample

^2^*CI* bootstrapped confidence intervals

**Table 8:** Spearman rank correlations for the LQ_0–28_ of the ILC-NOR self- and parent report

	M (SD) ILC-NOR	M (SD) parent ILC	Spearman’s rho	[95% CI]^2^		P
LQ_0–28_ CA ^1^ (N = 39)	22.57 (4.04)	22.03 (3.98)	− .038	− .577	.323	.819
LQ_0–28_ child (N = 24)	23.46 (4.08)	22.54 (3.41)	− .281	− .652	.190	.184
LQ_0–28_ adol. (N = 15)	21.13 (3.66)	21.20 (4.75)	.319	− .371	.757	.247

The Inventory of Life Quality in Children and Adolescents (ILC) QoL score (LQ_0–28_)

^1^*CA* children and adolescents—complete sample

^2^*CI* bootstrapped confidence intervals

### Usability

The DHH children and adolescents’ preferences for the presentation of the ILC are presented in Table 9.

**Table 9:** The DHH children and adolescents’ preferences for presentation of the ILC

	Frequency	Percent
ILC-NSL/comb.^1^ CA^2^	19	39.9
ILC-NOR CA	26	54.2
Do not know CA	3	6.3
Total	48	100.0
ILC-NSL/comb.^1^ child	10	47.6
ILC-NOR child	8	38.1
Do not know child	3	14.3
Total	21	100
ILC-NSL/comb.^1^ adol	9	33.3
ILC-NOR adol	18	66.7
Do not know adol	4	14.8
Total	27	100

The Inventory of Life Quality in Children and Adolescents (ILC)

^1^comb.: A potential combination of the written and signed versions

^2^*CA* children and adolescents—complete sample

During administration of the ILC-NSL and ILC-NOR, some of the children and adolescents commented that they spent more time completing the ILC-NSL because it took longer to view the video clips of the signed items than to read the items.

## Discussion

Internal consistency was established as good for both language and age versions. A comparison of the two language versions showed that the adolescent version corresponded closely for both item and total scores, whereas the child version did not correspond well between the languages. Construct validity based on PLS-SEM was found to be acceptable for the proposed one-factor model for both language versions and all ages.. This is also in line with the previously confirmed one-factor model based on the original theoretical concept of QoL that the ILC is based on [6, 7].

The ILC-NSL and ILC-NOR demonstrated similar psychometric properties to those reported for the ILC in other studies both for TH [6, 7] and DHH children and adolescents [1]. The ILC-NSL demonstrated the same pattern as the original Norwegian validation (ILC-NOR) with lower internal consistency based on Cronbach’s α for the child version than the adolescent version [6]. The relative cognitive immaturity in younger children or the significantly lower NSL skills may be a possible explanation for this.

Associations between the two language versions of the self-report were high for both item and scale scores for the ILC adolescent version. They were higher than we expected based on other studies comparing written and sign language versions of mental health assessments [23, 24]. This may indicate a close correspondence between the ILC-NSL and ILC-NOR because of equivalent phrasing in written Norwegian and NSL. Other reasons for the close correspondence may have been the high number of children and adolescents with a spoken language preference among this DHH sample or possibly good literacy, which was not assessed. The associations between the two language versions of the child self-report, however, were much weaker, indicating problems with the translation, literacy, or Norwegian sign language skills. As no DHH children or adolescents were included in the focus groups during the translation process, it is possible that the translation was not clear or not at an appropriate level for DHH children with varying NSL skills. Including them in the focus group, however, would have decreased the number of potential participants for this study. Literacy was not assessed in the current study; therefore, it is difficult to conclude on this matter. Other possible reasons for this finding might be that the child version is constructed for individual administration but was administered in groups in the current study. The individual administration is designed as a conversation with the child and contains longer sentences and explanations than the adolescent version. As the younger participants have attended deaf school less than the adolescents and their parents have received fewer sign language lessons, the children’s sign language skills might not enable them to cope with the longer sentences. Therefore, they might have benefitted from the adolescent version with its shorter and simpler sentences. Consequently, we suggest that a validation study be carried out for younger DHH children using the adolescent version of the ILC-NSL after having included DHH children in focus groups on this NSL version and making adjustments if necessary.

There was a moderate, but not significant, correlation between adolescent self-reports (ILC-NSL and ILC-NOR) and parent reports for QoL scores LQ_0–28_ whereas the two language versions of the child self-report showed no associations with the parent reports. This is somewhat in contrast to the significant, but low informant agreement reported previously [6] for TH children and adolescents, whereas other researchers on DHH child and adolescent QoL report similar low agreement with parent reports [2, 3, 49] as seen in our study. Pardo-Guijarro, Martínez-Andrés, Notario- Pacheco et al. [5], reason that hearing parents experience the impact of their children’s deafness on QoL to a larger degree than their children. Warner-Czyz, Loy, Roland et al. [2] argue that several aspects of QoL are less observable for parents, such as self-esteem, family, and friends. Others [4, 50] have suggested that DHH children and adolescents not sharing the same mode of communication with their parents might lessen the parents’ insight into their children’s subjective world, including QoL. Aanondsen, Jozefiak, Heiling et al. [31] find parent–DHH child correlations for the Strengths and Difficulties Questionnaire (SDQ) assessing mental health, close to those reported in another study [51] for TH children and adolescents. The difference in parent–child agreement between the SDQ and ILC might be related to the different nature of the items describing QoL compared with mental health symptoms (SDQ), which are more easily observed by others. This illustrates the definition of QoL as a subjective concept. The low agreement between parents and DHH, as well as TH children and adolescents, emphasizes the need to consider the self-report as the authentic QoL report, whereas the parent report should be used as supplemental information from a more remote informant [52]. This conclusion enhances the importance of developing sign language versions of generic QoL instruments for capturing DHH children and adolescents’ own views. This does not, however, lessen the importance of assessing parents’ perspective on their children’s QoL as is also emphasized by the authors of the ILC [6, 7].

Most of the DHH children and adolescents reported preferring the written instrument (ILC-NOR), and this preference was more pronounced for the adolescents than the children, possibly reflecting the lower NSL competence among children and their parent-reported preference for spoken Norwegian. There may have been subsamples based on spoken or sign language proficiency that could have influenced these results. These were not examined, however, due to the small sample size. Spontaneous feedback during administration indicated that the preference for the written version (ILC-NOR) was related to the less time-consuming nature of this version. Greater mastery of literacy in DHH adolescents could explain their preference for the written version of the ILC. The preference of the written version, however, is somewhat surprising given that other studies report reading difficulties to be frequent in many DHH children and adolescents [20, 22, 53] and their preference for sign language. As we only assessed spoken and sign language skills but not literacy, we could not test this.

### Strengths and limitations

A major strength of the current study is the use of a generic assessment tool for QoL that was translated into NSL, and that also examined psychometric properties for both written and sign language for DHH children and adolescents. A further strength of the choice of the ILC is the multi-informant perspective. Both these factors have been found necessary to solve some of the current inconsistencies in findings on the QoL of DHH children and adolescents.

A major limitation of the present study is the small sample size due to the limited number of signing DHH children and adolescents in the population. The sample size here was smaller than the minimum number of cases recommended for multivariate analyses based on covariance, especially when analyzing the child and the adolescent versions separately. This, in turn, poses a problem for a thorough psychometric evaluation of the ILC-NSL and ILC-NOR for DHH children and adolescents. Alternatively, the hypotheses could have been framed more precisely and tailored to the expected small sample size, in turn choosing statistical procedures more in line with these. By reporting the confidence intervals for the results, we have attempted to partly compensate for this. To offset the effects of small sample size, we have also used the PLS-SEM, which is known to be robust for such situations [42]. The combination of analyses used here was chosen as the best practical solution for the small sample size but leaves room for uncertainty regarding the conclusions.

A further limitation is the short interval of two to three days between the administration of the two language versions. This may have led to participants remembering their former answers and creating a bias. The randomized order of administration of the two versions was conducted to counteract this.

The lack of including the target population for the ILC-NSL in the focus group for the translation is a further limitation as well as the use of single-item measures to assess spoken and sign language skills which cannot be regarded as a complete assessment of the participants’ communication skills. A minor limitation is the absence of a gold standard for establishing convergent validity for QoL in DHH children and adolescents. The use of a written instrument, such as the KIDSCREEN, as a gold standard, however, would not have been reliable or valid because of the evidence showing that many DHH children and adolescents have reading difficulties [20, 22, 53] even though this did not seem prominent in our sample. Another translation cycle into NSL and validation of this translation would have been necessary and too time-consuming for the scope of the current study. A further limitation is the lack of test–retest reliability.

## Conclusion

The evaluation of the psychometric properties of the self-report ILC-NSL is promising. The use of the self-report ILC-NSL for assessing QoL in DHH children and adolescents is essential given its subjective nature. For children younger than the age of 11, the use of the ILC-NSL is more questionable, possibly because of their lower sign language skills. Until better alternatives are developed, we suggest that the psychometric properties of the written and NSL adolescent versions are studied for DHH children after focus groups are conducted, including representatives for the target population. Alternatively, that it is investigated whether individual rather than group administration may result in better usability and validity of the child ILC-NSL and ILC-NOR. Based on the children and adolescents’ feedback, we recommend presenting both the written and NSL versions in combination to evaluate QoL among DHH children and adolescents rather than using only one language. Further research on DHH children and adolescents is needed to solve the current inconsistencies in the findings related to QoL. Because of the small number of signing DHH children and adolescents in the population, cross-cultural studies should be encouraged; this would increase the possibility of conducting research on larger samples, as well as allowing for an examination of cross-cultural similarities and differences.

## Supplementary Information

**Additional file 1.** Appendix A and B.

## Data Availability

The datasets generated and analyzed during the current study are not publicly available because of the sensitivity of the information, as well as the small sample size. They are available from the local research committee or the corresponding author upon reasonable request.

## Abbreviations

QoL: Quality of life
DHH: Deaf and hard-of-hearing
TH: ILC
PedsQL: Pediatric Quality of Life Inventory
NSL: Norwegian Sign Language
KINDL: Kinder Lebensqualität Fragebogen
ILC: The Inventory of Life Quality
CAP: Categories of Auditory Performance
SIR: Speech Intelligibility Rating
SPS: Sign Language Production Scale
SUS: Sign Language Understanding Scale
CA: Children and adolescents—complete sample
CODA: Children of deaf adults
DG rho: Dillon–Goldstein’s rho
CI: Confidence interval
ICC: Intraclass correlation coefficients
CFA: Confirmatory factor analysis
WLSMV: Weighted least squares means and variances adjusted
RMSEA: Root mean square error of approximation
CFI: Comparative fit index
TLI: Tucker–Lewis index
PLS-SEM: Partial least squares structural equation modeling
AVE: Average variance extracted
