# Supplementary material for: Psychometric properties of the Inventory of Life Quality in children and adolescents in Norwegian Sign Language
Source: BMC Psychol. 2021 May 27;9:89. doi: 10.1186/s40359-021-00590-x (PMC8161577; doi:10.1186/s40359-021-00590-x)
Supplement: Supplementary file 1 — Additional file 1. Appendix A and B. [file 40359_2021_590_MOESM1_ESM.docx]

# Appendix A

**Table A** Mean gender differences (MD) with CIs for item scores for both ILC-NSL and ILC-NOR

|  | MD ILC-NSL | [95% CI] | | MD ILC-NOR | [95% CI] | |
| --- | --- | --- | --- | --- | --- | --- |
| LQ_0-28_ CA | .760 | -1.491 | 4.163 | -3.323 | -5.923 | -1.638 |
| School | -.102 | -.937 | .436 | .415 | .070 | .868 |
| Family | -.210 | -.912 | .231 | .330 | .009 | .567 |
| Other children | -.123 | -.909 | .414 | .446 | .035 | .848 |
| Alone | .057 | -.922 | .532 | .895 | .517 | 1.263 |
| Physical Health | .386 | -.040 | .980 | .497 | .088 | .977 |
| Mental Health | -.171 | -.961 | .405 | .269 | -.194 | .833 |
| Global QoL | -.595 | -1.019 | .121 | .471 | .018 | .901 |

*Notes.* CA: Complete sample MD: mean difference between boys and girls, CI: bootstrapped

# Appendix B

ILC-NSL complete sample (CA; N=49)

**Fig. B1** School **Fig. B2** Family

**

*Note.* Range of item scores 1–5, 1=high QoL

**Fig. B3** Other children **Fig. B4** Alone

**

*Note.* Range of item scores 1–5, 1=high QoL

ILC-NSL CA (N=49)

**Fig. B5** Physical Health **Fig. B6** Mental Health

**

*Note.* Range of item scores 1–5, 1=high QoL

**Fig. B7** Global QoL **Fig. B8** LQ_0-28_

**

*Note.* Range of item scores 1–5, 1=high QoL; QoL score (LQ_0-28_): range 0–28, 28=high QoL

ILC-NSL child (N=22)

**Fig. B9** School **Fig. B10** Family

**

*Note.* Range of item scores 1–5, 1=high QoL

**Fig. B11** Other children **Fig. B12** Alone

**

*Note.* Range of item scores 1–5, 1=high QoL

ILC-NSL child (N=22)

**Fig. B13** Physical Health **Fig. B14** Mental Health

**

*Note.* Range of item scores 1–5, 1=high QoL

**Fig. B15** Global QoL **Fig. B16** LQ_0-28_

**

*Note.* Range of item scores 1–5, 1=high QoL; QoL score (LQ_0-28_): range 0–28, 28=high QoL

ILC-NSL adolescent (N=27)

**Fig. B17** School **Fig. B18** Family

**

*Note.* Range of item scores 1–5, 1=high QoL

**Fig. B19** Other children **Fig. B20** Alone

**

*Note.* Range of item scores 1–5, 1=high QoL

ILC-NSL adolescent (N=27)

**Fig. B21** Physical Health **Fig. B22** Mental Health

**

*Note.* Range of item scores 1–5, 1=high QoL

**Fig. B23** Global QoL **Fig. B24** LQ_0-28_

**

*Note.* Range of item scores 1–5, 1=high QoL; QoL score (LQ_0-28_): range 0–28, 28=high QoL

ILC-NOR CA (N=56)

**Fig. B25** School **Fig. B26** Family

**

*Note.* Range of item scores 1–5, 1=high QoL

**Fig. B27** Other children **Fig. B28** Alone

**

*Note.* Range of item scores 1–5, 1=high QoL

ILC-NOR CA (N=56)

**Fig. B29** Physical Health **Fig. B30** Mental Health

**

*Note.* Range of item scores 1–5, 1=high QoL

**Fig. B31** Global QoL **Fig. B32** LQ_0-28_

**

*Note.* Range of item scores 1–5, 1=high QoL; QoL score (LQ_0-28_): range 0–28, 28=high QoL

ILC-NOR child (N=25)

**Fig. B33** School **Fig. B34** Family

**

*Note.* Range of item scores 1–5, 1=high QoL

**Fig. B35** Other children **Fig. B36** Alone

**

*Note.* Range of item scores 1–5, 1=high QoL

ILC-NOR child (N=25)

**Fig. B37** Physical Health **Fig. B38** Mental Health

**

*Note.* Range of item scores 1–5, 1=high QoL

**Fig. B39** Global QoL **Fig. B40** LQ_0-28_

**

*Note.* Range of item scores 1–5, 1=high QoL; QoL score (LQ_0-28_): range 0–28, 28=high QoL

ILC-NOR adol. (N=31)

**Fig. B41** School **Fig. B42** Family

**

*Note.* Range of item scores 1–5, 1=high QoL

**Fig. B43** Other children **Fig. B44** Alone

**

*Note.* Range of item scores 1–5, 1=high QoL

ILC-NOR adol. (N=31)

**Fig. B45** Physical Health **Fig. B46** Mental Health

**

*Note.* Range of item scores 1–5, 1=high QoL

**Fig. B47** Global QoL **Fig. B48** LQ_0-28_

**

*Note.* Range of item scores 1–5, 1=high QoL; QoL score (LQ_0-28_): range 0–28, 28=high QoL

# Appendix C

Construct validity – supplementary results

The ILC-NSL CA showed good model fit for χ2/df and acceptable model fit for two indices (CFI and TLI). The ILC-NOR CA showed acceptable fit for χ2/df and CFI. Factor loadings based on CFA were acceptable for all seven items of the ILC-NSL for adolescents. None of the modification indices for ILC-NSL or ILC-NOR suggested correlated residuals for the one-factor model. The results support the results of the PLS-SEM.

< Table C1 and C2 here>

**Table C1** Factor loadings, and model fit indices of the ILC-NSL based on CFA

| Items | *λ* (CFA) | *χ^2^(df)* | *p* | *χ^2^/df* | CFI | TLI | RMSEA | 90% CI RMSEA |
| --- | --- | --- | --- | --- | --- | --- | --- | --- |
| ILC CA (N=49) |  | 23.283 (14) | .056 | 1.663 | .947 | .920 | .116 | .000-.197 |
| School | .735 |  |  |  |  |  |  |  |
| Family | .815 |  |  |  |  |  |  |  |
| Other children | .680 |  |  |  |  |  |  |  |
| Alone (play/hobbies) | .388 |  |  |  |  |  |  |  |
| Physical Health | .260 |  |  |  |  |  |  |  |
| Mental Health | .609 |  |  |  |  |  |  |  |
| Global QoL | .725 |  |  |  |  |  |  |  |
| ILC child (N=22) |  | 17.543 (14) | .228 | 1.253 | .966 | .949 | .107 | .000-.245 |
| School | .914 |  |  |  |  |  |  |  |
| Family | .834 |  |  |  |  |  |  |  |
| Other children | .767 |  |  |  |  |  |  |  |
| Items | *λ* (CFA) | *χ^2^(df)* | *p* | *χ^2^/df* | CFI | TLI | RMSEA | 90% CI RMSEA |
| Alone | .279 |  |  |  |  |  |  |  |
| Physical Health | -.031 |  |  |  |  |  |  |  |
| Mental Health | .386 |  |  |  |  |  |  |  |
| Global QoL | .803 |  |  |  |  |  |  |  |
| ILC adol. (N=27) |  | 26.818 (14) | .020 | 1.916 | .903 | .854 | .184 | .071-.289 |
| School | .672 |  |  |  |  |  |  |  |
| Family | .912 |  |  |  |  |  |  |  |
| Other children | .534 |  |  |  |  |  |  |  |
| Alone | .664 |  |  |  |  |  |  |  |
| Physical Health | .616 |  |  |  |  |  |  |  |
| Mental Health | .692 |  |  |  |  |  |  |  |
| Global QoL | .618 |  |  |  |  |  |  |  |

*Notes.* The Inventory of Life Quality in Children and Adolescents (ILC); QoL score (LQ_0-28_);

CA: children and adolescents—complete sample

**Table C2** Factor loadings, and model fit indices for the ILC-NOR based on CFA

| Items | *λ* (CFA) | *χ^2^(df)* | *p* | *χ^2^/df* | CFI | TLI | RMSEA | 90% CI RMSEA |
| --- | --- | --- | --- | --- | --- | --- | --- | --- |
| ILC CA (N=56) |  | 48.086 (14) | .001 | 3.435 | .928 | .892 | .209 | .146-.275 |
| School | .805 |  |  |  |  |  |  |  |
| Family | .632 |  |  |  |  |  |  |  |
| Other children | .714 |  |  |  |  |  |  |  |
| Alone (play/hobbies) | .448 |  |  |  |  |  |  |  |
| Physical Health | .446 |  |  |  |  |  |  |  |
| Mental Health | .940 |  |  |  |  |  |  |  |
| Global QoL | .862 |  |  |  |  |  |  |  |
| ILC child (N=25) |  | 15.658 (14) | .335 | 1.118 | .990 | .986 | .069 | .000-.0211 |
| School | .832 |  |  |  |  |  |  |  |
| Family | .506 |  |  |  |  |  |  |  |
| Other children | .863 |  |  |  |  |  |  |  |
| Items | *λ* (CFA) | *χ^2^(df)* | *p* | *χ^2^/df* | CFI | TLI | RMSEA | 90% CI RMSEA |
| Alone (play/hobbies) | .547 |  |  |  |  |  |  |  |
| Physical Health | .369 |  |  |  |  |  |  |  |
| Mental Health | .837 |  |  |  |  |  |  |  |
| Global QoL | .887 |  |  |  |  |  |  |  |
| ILC adol. (N=31) |  | 37.153 (14) | .007 | 2.654 | .919 | .878 | .231 | .142-.323 |
| School | .718 |  |  |  |  |  |  |  |
| Family | .803 |  |  |  |  |  |  |  |
| Other children | .543 |  |  |  |  |  |  |  |
| Alone (play/hobbies) | .617 |  |  |  |  |  |  |  |
| Physical Health | .390 |  |  |  |  |  |  |  |
| Mental Health | .952 |  |  |  |  |  |  |  |
| Global QoL | .875 |  |  |  |  |  |  |  |

*Notes.* The Inventory of Life Quality in Children and Adolescents (ILC); QoL score (LQ_0-28_);

CA: children and adolescents—complete sample
